# Patterns of postpartum contraceptive use among Somali immigrant women living in Minnesota

**DOI:** 10.1186/s40834-017-0041-x

**Published:** 2017-04-04

**Authors:** Amy Millar, Rachel Isaksson Vogel, Sabrina Bedell, Maureen Ayers Looby, Jessica L Hubbs, Bernard L. Harlow, Rahel Ghebre

**Affiliations:** 1 University of Minnesota Department of Obstetrics, Gynecology and Women’s Health, 420 Delaware St SE, MMC 395, Minneapolis, MN 55455 USA; 2 Boston University School of Public Health, Boston, MA USA; 3 Human Resources for Health Program Rwanda and Yale School of Medicine, New Haven, CT USA

**Keywords:** Contraceptive counseling, Family planning service provision, Intrauterine device, Long-acting reversible contraception

## Abstract

**Background:**

The postpartum period is a crucial time to provide family planning counseling and can decrease incidence of adverse reproductive outcomes. The purpose of this study was to characterize patterns of postpartum contraception and to investigate long acting reversible contraceptive (LARC) use among Somali women living in a metropolitan area of Minnesota in an effort to provide better family planning and reproductive health counseling in this growing immigrant population.

**Methods:**

A retrospective chart review was conducted of Somali women who delivered between January 1, 2011 and December 31, 2014. Information was collected regarding family planning counseling provided and contraceptive methods chosen at the postpartum clinic visit.

**Results:**

Of the 747 Somali women who delivered during this time period, 56.4% had no postpartum follow up visit. At the postpartum visit, 88.3% of women received family planning counseling and 80.8% chose a contraceptive method with the remainder declining. The intrauterine device (IUD) was the most popular contraceptive method, chosen by 39.7% of women. Other than parity, no statistically significant differences were observed between women who chose LARC versus other contraceptive methods. Of the women that chose a LARC, 39.4% had it placed at the time of their postpartum visit; immediate placement was statistically significantly more likely with more recent delivery, lower BMI and obstetrician as the provider type.

**Conclusions:**

The IUD was the most popular method of postpartum contraception. There was a trend toward increase in LARC use with increasing parity. Same-day LARC placement was uncommon, but should be encouraged in this population given high loss to follow up rate.

## Background

The postpartum period is a crucial time to provide family planning counseling. Opportunities to address contraception are plentiful during this time period and a discussion surrounding family planning can serve to reduce the incidence of short interpregnancy interval [1, 2]. Given that a short interpregnancy interval is associated with both adverse maternal and neonatal outcomes [3], providing reliable and timely contraception in the postpartum period can support adequate spacing between pregnancies and thus decrease incidence of adverse pregnancy outcomes. In a patient with regular prenatal care, contraceptive counseling should begin during the patient’s antenatal course and continue to be discussed throughout the pregnancy and after delivery. A variety of contraceptive methods are considered safe in the immediate postpartum period and may be initiated immediately after delivery [4]. By the traditional six week postpartum visit, there are no absolute contraindications to any available methods of contraception in the average risk woman [4].

The use of long acting reversible contraceptives (LARCs) has increased over the past decade in the United States [5, 6]. The American College of Obstetricians and Gynecologists and the Centers for Disease Control support the use of LARC in the immediate postpartum period, including placement at the time of delivery [6]. Not only is use of LARC considered safe during this postpartum time period, but LARC methods have been shown to be superior to shorter-acting methods in efficacy, rate of discontinuation and incidence of unintended pregnancy [6]. Studies of recent trends in LARC use in the United States show a higher rate of use in the subgroup of women born outside of the United States [5].

Minnesota is home to the largest population of Somali immigrants and refugees in the United States [7]. Few studies have explored the use of contraception in the Somali population; those that have focused on eliciting the attitudes and perceptions of small groups of Somali refugees living outside the United States [8, 9]. There is a paucity of available data regarding the most common current contraceptive practices of the Somali population living in the United States. In our Somali patient population we have observed an above average incidence of short interpregnancy interval and grand multiparity. Given the potential for increased adverse pregnancy outcomes in the setting of grand multiparity [10, 11] and closely spaced pregnancies [3, 12], it is important to provide appropriate family planning counseling to this at-risk population in the postpartum period.

The number of African refugees living in the United States is expected to continue rising [13, 14], and an adequate understanding of contraceptive use in this population is necessary to help guide patient care. The purpose of this study was to characterize patterns of contraceptive use among the Somali immigrant patient population in the postpartum period. Additionally, as LARC use has been increasing in the United States in recent years [5, 6], we sought to determine to what extent, if any, this same trend of LARC use has been reflected among the population of Somali patients within our practice.

## Methods

A retrospective chart review of the electronic medical records (EMR) of women greater than 18 years old who self-identified as Somali ethnicity and delivered at the University of Minnesota between January 1, 2011 and December 31, 2014 was conducted. The study was reviewed and approved by the Institutional Review Board at the University of Minnesota. Somali women were identified who delivered with either obstetrics (OB), certified nurse midwife (CNM) or family practice (FP) providers. Patients without a documented postpartum visit within 90 days of delivery with one of the above provider types were excluded. The EMR was reviewed and demographic and clinical data associated with the most recent delivery during the study period were abstracted. For patients with multiple deliveries within the study period, only data pertaining to the most recent delivery were recorded.

The outcomes of interest for this study were whether contraception was chosen at the postpartum visit, whether a LARC was chosen, and among those who chose LARC, whether it was placed at the post-partum visit. Independent variables of interest included year of delivery, marital status (single, married/partnered, divorced/separated), provider type (CNM, FP, OB), mother age at delivery (continuous), body mass index (kg/m [2]; continuous), and parity (continuous).

Demographic and pregnancy characteristics, along with data relating to postpartum visits and documented contraception choice of postpartum Somali women were summarized using descriptive statistics. Among women who chose a contraception method at the time of their postpartum visit, the associations between demographic and provider characteristics and contraceptive method choice (LARC vs. Other) were conducted using Chi-squared and Fisher’s Exact tests as appropriate for categorical variables and t-tests and Wilcoxon rank-sum tests as appropriate for continuous variables. Similarly, among those who decided to use LARCs, comparisons of demographic and provider characteristics were conducted between those who did and did not have a LARC placed at the time of their postpartum visit. All analyses were performed using SAS version 9.3 (Cary, NC). As this was an exploratory analysis no adjustment was made for multiple comparisons and *p*-values <0.05 were considered statistically significant.

## Results

**Fig. 1:**
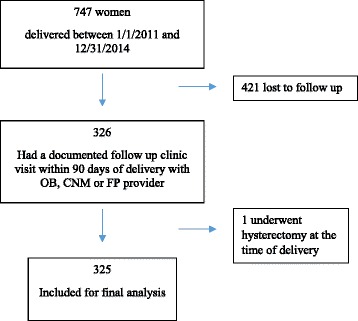
Flow diagram of included patients

We identified 747 distinct Somali women with at least one delivery during our 5 years study period; 224 (30.0%) delivered by cesarean section and 523 (70.0%) delivered vaginally. Of these 747 women, 326 (43.6%) had a documented follow up visit within 90 days of delivery with either an OB, CNM or FP provider within the same health system; 421 (56.4%) did not and thus were considered lost to follow up in the postpartum period. One had a planned cesarean hysterectomy and was therefore excluded from final analysis (Fig. 1). When comparing women who did follow-up with those who did not follow-up, Somali women who had a cesarean section were more likely to follow-up than those who had a vaginal birth (49.1% vs. 40.5%; *p* = 0.03) and accordingly, those who were delivered by an MD were more likely to return (45.1% vs. 33.1%; *p* = 0.01). There were no differences between those who did and did not follow-up by age, primary language, or parity.

A total of 325 (43.6%) women were included in further analyses. The mean age was 30.6 ± 5.7 years at delivery. Most were married or partnered (281, 86.5%) and the median parity was 4 (Table 1).

**Table 1:** Demographic and pregnancy characteristics (*N* = 325)

Variable	Number	Percent
Year of Delivery		
2011	42	12.9
2012	82	25.2
2013	91	28.0
2014	110	33.9
Interpreter Needed		
No	72	30.6
Yes	163	69.4
Marital Status		
Single	38	11.7
Married/Partnered	281	86.5
Divorced/Separated	6	1.8
Family planning counseling provided prior to hospital discharge		
No	58	17.9
Yes	267	82.2
Contraceptive method selected at time of delivery		
Yes	115	
No- Declined	66	24.7
Undecided	86	32.2
*N/A- not discussed*	*58*	
Contraception chosen at time of delivery, prior to hospital discharge		
Intrauterine device	66	24.7
Etonogestrel implant	13	4.9
Barrier	10	3.8
Abstinence	9	3.4
Combined oral contraceptive pills	8	3.0
Natural family planning	4	1.5
Permanent sterilization	3	1.1
Progesterone only contraceptive pills	2	0.8
Mother age at delivery, mean ± SD, years	325	30.6 ± 5.7
BMI, mean ± SD, kg/m^2^	320	28.2 ± 5.3
Parity, median (range)	325	4 (1–10)

*Abbreviations*: *IUD* Intrauterine device, *SD* Standard deviation, *BMI*

Postpartum visits were conducted by a variety of providers, with generally even distribution between OB, FP and CNM (Table 2). A total of 287 (88.3%) women received family planning counseling at their postpartum visit and of these, 232 (80.8%) chose a method of contraception; the remaining 55 declined contraception. A LARC method was chosen by 104 (44.8%) Somali women. The most popular contraceptive method chosen by this population was the IUD, with 92 (39.7%) women choosing this option.

**Table 2:** Details of postpartum visit

Variable	Number	Percent
Provider Type		
CNM	100	30.8
FP	101	31.1
OB	124	38.2
Family planning counseling provided at postpartum visit		
Yes	287	88.3
No	38	11.7
Contraceptive method selected at postpartum visit		
Yes	232	80.8
No- Declined	55	19.2
*N/A- not discussed*	*38*	
Contraception type selected at postpartum visit		
Intrauterine device	92	39.7
Etonorgestrel implant	12	5.2
Barrier	32	13.8
Abstinence	20	8.6
Combined oral contraceptive pills	16	6.9
Natural family planning	10	4.3
Permanent sterilization	0	0.0
Progesterone only contraceptive pills	34	14.7
Depot medroxyprogesterone acetate	15	6.5
Combined hormonal patch	1	0.4
Contraception type selected at postpartum visit—LARC vs Other		
LARC	104	44.8
Other	128	55.2

*Abbreviations*: *CNM* Certified Nurse Midwife, *FP* Family Practitioner, *OB* Obstetrician, *LARC* Long acting reversible contraception

Comparisons of patients by whether they chose or declined contraception at the postpartum visit indicated no statistically significant differences based on year of delivery, marital status, parity, provider type or receipt of family planning counseling while in the hospital.

Patients with greater parity were more likely to choose LARCs than those who did not (median 4 vs 3, *p* = 0.02). There were no other statistically significant differences by year of delivery, marital status, provider type, maternal age or BMI as compared to those that chose non-LARC methods (Table 3). Similarly, patients who received family planning counseling prior to hospital discharge were no more likely to choose LARC as compared to other contraceptive methods.

**Table 3:** Comparison of demographic and provider characteristics between patients selecting LARC vs other contraceptive methods at the postpartum visit

	Did Not Select		Selected		*p*-value
	LARC		LARC		
	*N* = 128		*N* = 104		
Variable	N	%	N	%	
Year of Delivery					0.95
2011	18	14.	12	11.5	
2012	32	125.0	27	26.0	
2013	36	28.1	31	29.8	
2014	42	32.8	34	32.7	
Marital Status					0.25
Single	15	11.7	7	6.7	
Married/Partnered	109	85.2	96	92.3	
Divorced/Separated	4	3.1	1	1.0	
Family planning counseling provided prior to hospital discharge					0.53
No	25	19.5	17	16.4	
Yes	103	80.5	87	83.7	
Provider Type					0.66
CNM	45	35.4	34	32.7	
FP	34	26.8	25	24.0	
OB	48	37.8	45	43.3	
Mother age at delivery, mean ± SD, years	128	30.4 ± 5.4	104	30.5 ± 6.0	0.98
BMI, mean ± SD, kg/m^2^	126	27.8 ± 4.5	104	29.0 ± 5.9	0.10
Parity, median (range)	128	3 (1–10)	104	4 (1–10)	0.02

*Abbreviations*: *LARC* Long acting reversible contraception, *CNM* Certified Nurse Midwife, *FP* Family practitioner, *OB* Obstetrician, *SD* Standard deviation, *BMI* Body mass index

Among the 104 patients who chose a LARC at their postpartum visit, 41 (39.4%) had the LARC placed during that visit. For those for whom the LARC was not placed at the postpartum visit, the most commonly cited reasons for delaying placement were either the specific provider did not perform LARC placement procedures or the provider preferred the patient return to clinic in 2 weeks. Patients were significantly more likely to have the LARC placed at the time of their postpartum visit if they delivered more recently (*p* = 0.007), had a lower BMI (*p* = 0.05) or had their postpartum visit with an obstetrician as compared to CNM or FP provider (*p* = 0.01). Among the 63 patients that desired LARC but did not have it placed at their postpartum visit, 40 (63.5%) returned within the subsequent three months for placement; the remaining 23 did not.

## Discussion

This study population provides a unique opportunity to evaluate contraceptive use among immigrant Somali women in America. Our findings demonstrated that while Somali patients use a variety of contraception in the postpartum period, LARC—specifically, the copper IUD—is the most commonly chosen contraceptive method. The remaining contraceptive choices were fairly evenly distributed among a variety of methods. A prior study of Somali refugee women living in Finland similarly reported the IUD to be the most popular contraceptive method in that population, though overall contraceptive use was low with only 12 of 67 (17.9%) women using the IUD and 18 of 67 (26.8%) women surveyed reporting any contraception use [8]. This study did not report women’s rationale for choice of contraceptive method. Further studies are needed in our patient population to understand the predominance of IUD use. Possible reasons include patient desire for a hormone-free option, familiarity of this contraceptive method within the community, and provider preferential counseling on this method. There was no difference in LARC use between women who did and did not receive contraceptive counseling in the hospital, which may suggest that attitudes toward a particular contraceptive method are formed prior to the time of delivery. Supporting this, our study showed that when women were questioned in the hospital, the IUD was similarly the most commonly chosen method.

Missed opportunity for contraceptive counseling and management was identified as over half of patients failed to come for a documented follow-up within 90 days of their delivery, indicating a disparity in postpartum care among this Somali immigrant population. In contrast, the rate of postpartum follow up nationally is estimated at 89% [15]. Not surprisingly, Somali women were more likely to attend a postpartum visit if they delivered by cesarean section, likely due to the increased complexity of recovery from this procedure

Although nearly 20% of our patients declined contraception, this number was much lower than previous studies suggesting the majority of Somali women use no contraception [8, 9]. However, this does not account for the large group of patients in our study who were lost to follow up. Previous studies in this patient population have focused on the attitudes and perceptions of small groups of Somali women living outside of the United States. These studies have suggested a variety of barriers to contraceptive use among Somali immigrant women including misinformation, partner preference against contraception, mistrust of Western medicine, language barrier, religious beliefs, and practical need for a large family [8, 9]. Our study suggests that the attitudes and perceptions of Somali women toward contraception may be altering given the increased frequency of contraceptive use. Additionally, previous studies show that Somali women living outside of Somalia express difficulty caring for a large number of children without available family support [8], which likewise may have played a role in some women in our study desiring long acting contraception.

We did find an increase in LARC use with increasing parity, which is consistent with previous studies among the general United States population [5]. More information is needed on the attitudes and motivations of this immigrant patient population in order to draw conclusions about the specific role of parity in choice of a LARC. A trend towards increasing use of LARC methods over time was not reflected in our patient population over a period of four years, in contrast to recent studies at the national level [5]. Previous studies show that the increase in prevalence of LARC use is most notable among the nulliparous patient population [5], possibly a reflection of the changes made to United States Food and Drug Administration labeling and Centers for Disease Control recommendations in recent years supporting the use of IUDs in the nulliparous patient population [6]. A study that includes nulliparous Somali patients would help to identify whether this same trend persists in this patient population.

Of the women who chose a LARC at the postpartum visit, less than half had the LARC placed in the same visit and ultimately one fifth did not have it placed at all. Despite the fact that a required return visit is known to act as a barrier to LARC placement [16], previous studies have shown that same-day placement is not common practice [17, 18], which was consistent with our findings. Cited reasons in the literature for requiring multiple visits include the time constraint at a single visit, the desire to wait for infectious testing to return, and the hope of decreasing discontinuation if patients spend more time contemplating choice of method [17]. Given the high loss to follow up rate in this patient population, same-day LARC placement in patients who desire this contraceptive method is essential.

Beyond same-day LARC placement, immediate postpartum placement of LARC (i.e. prior to hospital discharge) should be offered in this patient population. Immediate postpartum LARC placement is safe, cost-effective and results in higher rates of IUD use at 6 months postpartum as compared to delayed placement [19, 20]. Immediate postpartum LARC placement is endorsed by the American Congress of Obstetrics and Gynecology, the American College of Nurse-Midwifes, the American Academy of Family Physicians and the Society for Maternal-Fetal Medicine, among other professional organizations [19]. Ideally, contraceptive counseling should begin in the antenatal period and can include the option of immediate postpartum LARC. Our study did not address antenatal contraceptive counseling, however, it is customary in all of our clinics to provide contraceptive counseling throughout pregnancy. In our hospital at the time of this study, patients were not routinely offered immediate postpartum LARC. Currently, LARC devices are stocked on the labor and delivery unit at our hospital, though barriers to reimbursement do exist and ongoing advocacy is needed to revise state-dictated repayment [21]. However, given the high loss to follow up rate in this population, it is crucial to offer immediate postpartum LARC placement. Further studies are necessary to investigate the acceptability of immediate postpartum LARC placement in this population of women.

There are limitations to this study. First, due to the retrospective nature of this study, our results are subject to selection and information bias due to the reliance on the EMR data. Second, there was significant loss to follow-up in this population and therefore our results may not be generalizable to all Somali immigrant women. Finally, we have limited data on the long term use of LARCs and discontinuation rates in this patient population. Recognizing these limitations, findings from this study represents a significant step in understanding contraception choices within this immigrant population. Future studies are needed to determine risk factors for failure to follow up among this patient population and prospective efforts to improve compliance.

## Conclusions

Our study was the first to characterize the postpartum contraceptive practices of the Somali immigrant population in the state of Minnesota. We examined a large number of women over several years to draw conclusions about the most effective means to provide family planning counseling and care to this group of women. We found a high loss to follow up rate, necessitating better provision of same-day contraception to this group. Among women that did follow up, we found a high rate of contraception use, with many preferring to use the intrauterine device. The information reported in this study represents an important first step toward providing better family planning services to a large immigrant population in the United States.

## Abbreviations

BMI: Body mass index
CNM: Certified Nurse Midwife
FP: Family practitioner
LARC: Long acting reversible contraception
OB: Obstetrician
SD: Standard deviation.
